# D-dimer levels to exclude pulmonary embolism and reduce the need for CT angiography in COVID-19 in an outpatient population

**DOI:** 10.1371/journal.pone.0297023

**Published:** 2024-01-17

**Authors:** Anita Kovács, Dóra Hantosi, Nikoletta Szabó, Annamária Letoha, Csaba Lengyel, Imre Földesi, Katalin Burián, András Palkó, Dániel Veréb, Zsigmond Tamás Kincses

**Affiliations:** 1 Department of Radiology, Albert Szent-Györgyi Medical Center, Albert Szent-Györgyi Medical School, University of Szeged, Szeged, Hungary; 2 Department of Neurology, Albert Szent-Györgyi Medical Center, Albert Szent-Györgyi Medical School, University of Szeged, Szeged, Hungary; 3 Department of Internal Medicine, Albert Szent-Györgyi Medical Center, Albert Szent-Györgyi Medical School, University of Szeged, Szeged, Hungary; 4 Department of Laboratory Medicine, Albert Szent-Györgyi Medical Center, Albert Szent-Györgyi Medical School, University of Szeged, Szeged, Hungary; 5 Department of Medical Microbiology, Albert Szent-Györgyi Medical Center, Albert Szent-Györgyi Medical School, University of Szeged, Szeged, Hungary; Heidelberg University Hospital Department of Anaesthesiology: Universitatsklinikum Heidelberg Klinik fur Anasthesiologie, GERMANY

## Abstract

**Objectives:**

Emerging results indicate that, in COVID-19, thromboembolic complications contribute to the high mortality and morbidity. Previous research showed that the prevalence of pulmonary embolism (PE) is between 25–50% in COVID-19 patients, however, most of these reports are based on data from patients with severe pneumonia, treated in intensive care units.

**Materials and methods:**

We conducted a retrospective, single-center, observational study to estimate the prevalence of PE in COVID-19 patients who underwent CT angiography and to identify the most important predictors.

Adult outpatients with COVID-19, who presented at our COVID Outpatient Clinic between 1^st^ and 31^st^ of March in 2021 and underwent CTA examination were included in this study. Multiple linear regression analysis was used to identify predictors of PE in COVID-19 patients. The predictors were: age, gender, disease duration, CT severity index and log-transformed quantitative D-dimer (logQDDIM) value.

**Results:**

843 COVID-19 patients were included into the study. 82.56% (693 patients) of the infected patients had a pulmonary CTA examination and D-dimer levels (mean age: 59.82 years ± 15.66). 7.61% (53 patients) of the patients had PE. 2.02% (14 patients) of the patients had main branch or lobar PE.

The multiple regression analysis found that only logQDDIM was a significant predictor. A logQDDIM cut-off value of 0.0169 (1.0171 ug/ml serum D-dimer) predicted PE with 99% sensitivity (p<0.0001, degree-of-freedom = 570, AUC = 0.72).

**Conclusions:**

We demonstrated in a large cohort of COVID-19 patients that a cut-off value of QDDIM of 1ug/ml can exclude pulmonary embolism in an outpatient setting, implicating that QDDIM might potentially supersede CTA as a screening approach in COVID-19 outpatient clinics.

## Introduction

The mortality and morbidity of COVID-19 are high and emerging results indicate that a significant proportion of these cases are related to thromboembolic complications, in particular pulmonary embolism (PE). Reports indicate that PE occurs in COVID-19 patients with a prevalence of 25–50% [1,2] and PE is associated with higher mortality [3].

Chest computer tomography (CT) is a sensitive and specific approach to detect COVID-19 and identify the extent of lung involvement [4]. CT angiography (CTA) is the gold standard method for detecting PE in patients with high pre-test probability. Consequently, CT and CTA are increasingly used in the diagnostic workup of COVID-19 patients. While the excellent performance of these approaches is unquestionable, their use as a screening test might not be warranted because of radiation exposure and the risk of adverse reactions to contrast agents. It is crucial to narrow the patient population and use CTA in as a diagnostic tool only if the probability of PE is high.

Several studies showed that PE is common in COVID-19 patients, however, most of these reports concern patients with severe pneumonia, the vast majority of whom were treated in intensive care units [1–3,5,6].

The clinical symptoms of COVID-19 and PE overlap significantly and, regarding the overwhelming number of patients presenting to COVID outpatient clinics, a screening tool for PE could be valuable. However, CTA, which uses contrast material and relatively large radiation doses, could only be a screening approach if the prevalence of PE in an outpatient setting was also high and there were no other, less invasive alternatives. Up to now, only a few publications investigated the prevalence of PE in COVID-19 outpatients [7–12]. As a molecular biomarker, serum D-dimer levels are a good predictor of PE [13–18], but in COVID-19, the pathomechanism of thromboembolic complications might be different [19,20], hence the same cut-off values of serum D-dimer might not be applicable.

Therefore, we conducted a retrospective, single-center, observational study to estimate the prevalence of PE in COVID-19 patients and identify its most important predictors. Our final aim was to identify predictors and cut-off values that best determine which patients should be selected for CTA.

## Materials and methods

### Subjects

In this retrospective, single centre, observational study, SARS-CoV-2 infected adult (>18 years of age) outpatients were included who presented at the COVID Outpatient Clinic of the Szent-Györgyi Albert Clinical Center, University of Szeged between 1^st^ and 31^st^ of March in 2021 and had CTA examination.

During the studied period all patients with upper respiratory symptoms were referred to the COVID Outpatient Clinic of the Szent-Györgyi Albert Clinical Center irrespective of the severity of the symptoms. All patients had rapid antigen test to identify SARS-CoV-2 infection. If this test negative, but infection was still suspected, PCR test was performed (for details see below). All patients had D-dimer level measured. Due to experiences gained from isolated cases, CTA was increasingly requested around this time for COVID-19 patients irrespective of symptom severity or D-dimer levels. The aim of the current study was to identify the prevalence and predictors of PE in order to reduce these unnecessary CTA examinations.

This study was approved by the Human Investigation Review Board of the University of Szeged (ref no.: 4997). In view of the retrospective nature of the investigation, the committee granted waiver from providing signed consent forms.

The data cannot be publicly shared as per the ethics committee’s decision, which was based on the granted waiver exempting the need for signed consents. Data are available from the local ethics committee (contact via mail: Human Investigation Review Board of the University of Szeged H-6720 Dugonics square 13. 1^st^ Fl.33, or email: office.rkeb@med.u-szeged.hu) for researchers who meet the criteria for access to confidential data.

### Laboratory tests

Highly sensitive quantitative D-dimer levels were measured by an immunoturbidimetric method using a Stago StarMax-3 analyser (Biomedica Hungária Kft. Budapest, Hungary). The limit of detection was 0.27μg/ml as stated by the manufacturer. Two concentrations were applied (0.69 microgram/mL and 2.3 microgram/mL) for the evaluation of the precision. The intraassay CV% was 6.6 and 2.4, respectively. The interassay CV% was 4.6 and 1.2, respectively. The intralaboratory CV% was 7.3 and 3.2, respectively.

To verify SARS-CoV-2 infection, the COVID-19 Ag RAPID TEST DEVICE (Abbott Rapid Diagnostics Jena GmbH, Jena, Germany) antigen test was used from nasopharyngeal swabs. In patients whose Antigen test (Ag test) was negative, but who otherwise exhibited typical symptoms or came into close contact with an infected individual, the Fosun COVID-19 RT-PCR Detection Kit (Fosun Pharma, Shanghai, China) was used from nasopharyngeal swabs.

### CT scanning

CT pulmonary angiographies were performed on a 64-slice GE Revolution Evo scanner (GE Healthcare, Chicago, IL, USA). The scan area extended from diaphragm to lung apex. CTAs were acquired in helical mode, axial slices of 0.625mm were reconstructed, with tube voltage of 100/120 kVP and tube current of 200/500 mA depending on the body weight of the patient.

All patients received 50–60 ml contrast agent of 370mg/ml Untravist (iopromide), followed by 20ml saline administered with a flow rate of 4ml/s through an 18-gauge cannula placed in the antecubital vein. Scan timing was individualised using bolus tracking with a threshold of 120 Hounsfield units in the pulmonary trunk.

Reporting was performed on an eRad PACS system (version 8.1, Greenville SC, USA), on Eizo Radiforce RX850 displays (Hakusan, Ishikawa, Japan). PE was evaluated on the CTA images and was categorised to main branch, lobar, segmental and sub-segmental PE. A scoring system simplified from [21] was used to describe the lung involvement by COVID-19 pneumonia on the CT images (CT severity index: CTSI). The lungs were visually scored from 0 to 5 as: 0: no sign of COVID-19, 1: <5% involvement, 2: 6–25% involvement, 3: 26–50% involvement, 4: 51–75% involvement, 5: >75% involvement. The lobes were not judged separately, one score was given for the entire chest.

Twenty-one attending radiologists with 5 years training before board examination and further 2–40 years of experience evaluated the CT(A) scans. Readers were unaware of the D-dimer levels at the time of reporting. Other clinical information (such as complaints, clinical symptoms, previous diseases, medication) were available for the radiologists.

### Data analysis

Age, gender, duration of symptoms at presentation, D-dimer levels and CT severity index were recorded for all patients. Data was anonymised during data collection.

IBM SPSS Statistics for Mac (Armonk NY: IBM Corp) and Matlab (Mathworks Inc, Natick, Massachusetts) were used for statistical analysis. Continuous variables were compared using Student’s t-test if the distribution was found normal based on the Kolgomorov-Smirnov test. Pearson correlations were calculated to investigate the associations between various variables.

Multiple logistic regression was used to identify the demographical, clinical and laboratory parameters that could predict PE in SARS-CoV-2 infected patients. Only cases with a complete dataset were included in the analyses. Sensitivity and specificity were calculated for each predictor.

## Results

**Fig 1:**
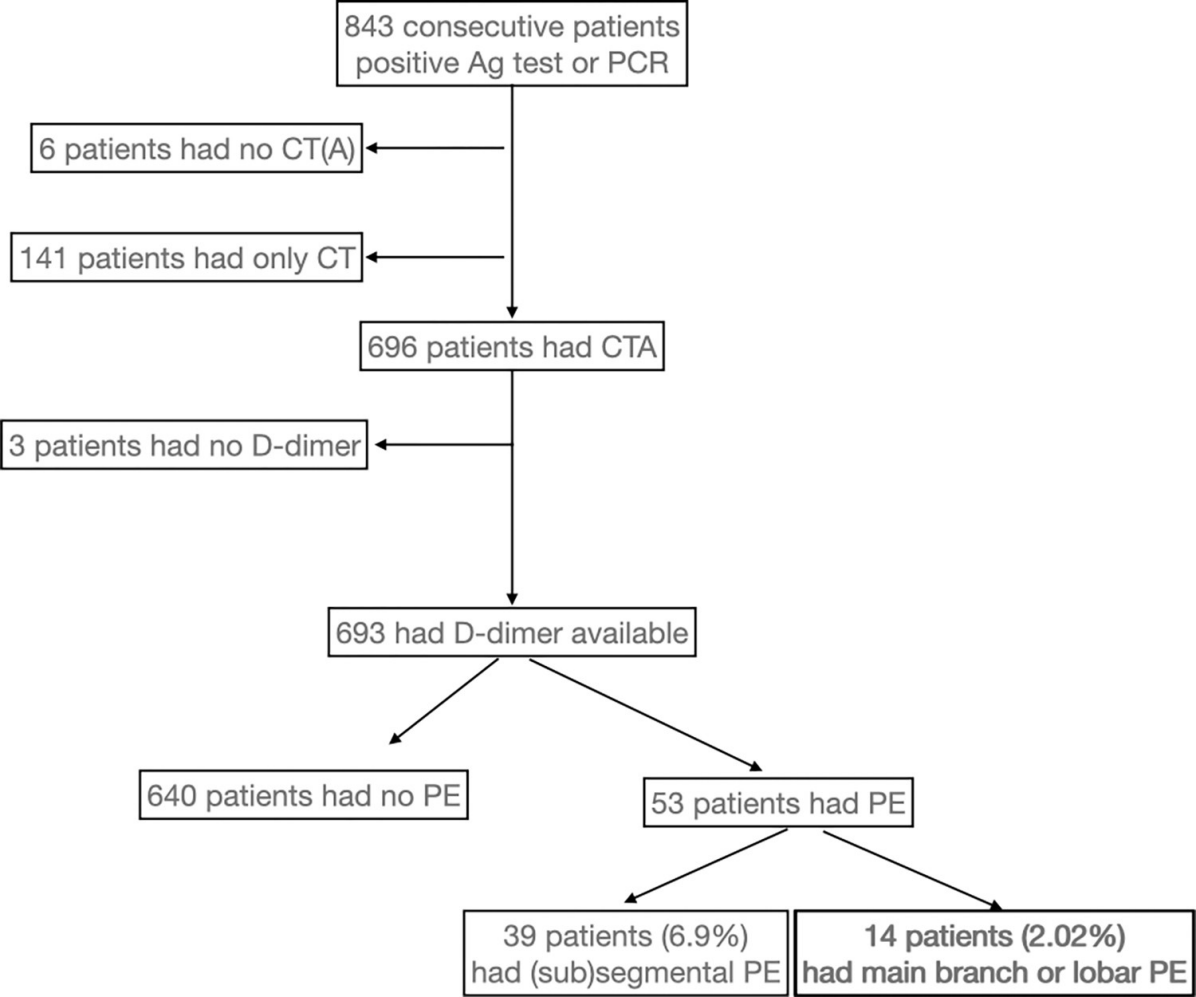
Study flowchart. PCR: Polymerase chain reaction test. Ag: Antigen test. (Sub)segmental stands for segmental and subsegmental emboli.

Between 1^st^ and 31^st^ of March in 2021, 843 COVID-19 patients attended the COVID Outpatient Clinic who had positive PCR or Ag test confirming SARS-CoV-2 infection. 82.56% of the infected patients had a pulmonary CTA examination. 7.61% of these patients had PE (Fig 1) and 2.02% had lobar or main branch embolus (the rest had segmental and subsegmental embolism). The clinical characteristics of the patients are summarised in Table 1.

**Table 1:** Clinical characteristics of the patients.

Clinical characteristics				
	Patients who had CTA and D-dimer	Patients with no PE	Main branch and lobar PE	(Sub)segmental PE
n	693	640	14	39
Age (years)	59.82±15.66	59.22±15.6*	66.78±14.39*	67.02±14.94*
Male	366 (52.81%)	336 (52.5%)	8 (1.15%)	22 (44.8%)
CTSI	2.3±1.3	2.27±1.29	2.64±1.49	2.69±1.28
Disease duration (days)	6.58±4.05	6.67±4.08	5.83±3.76	4.82±3.18

The age of the patients who had PE was significantly higher than the age of non-PE patients. Disease duration is the time since symptom onset(*). CTSI: CT severity index. Disease duration: Time since the onset of symptoms to the CTA scan.

### Predictors of pulmonary embolism

Serum D-dimer concentrations exhibited a non-normal distribution, therefore the D-dimer concentration was normalised by taking the natural logarithm of the concentration (logQDDIM).

**Fig 2:**
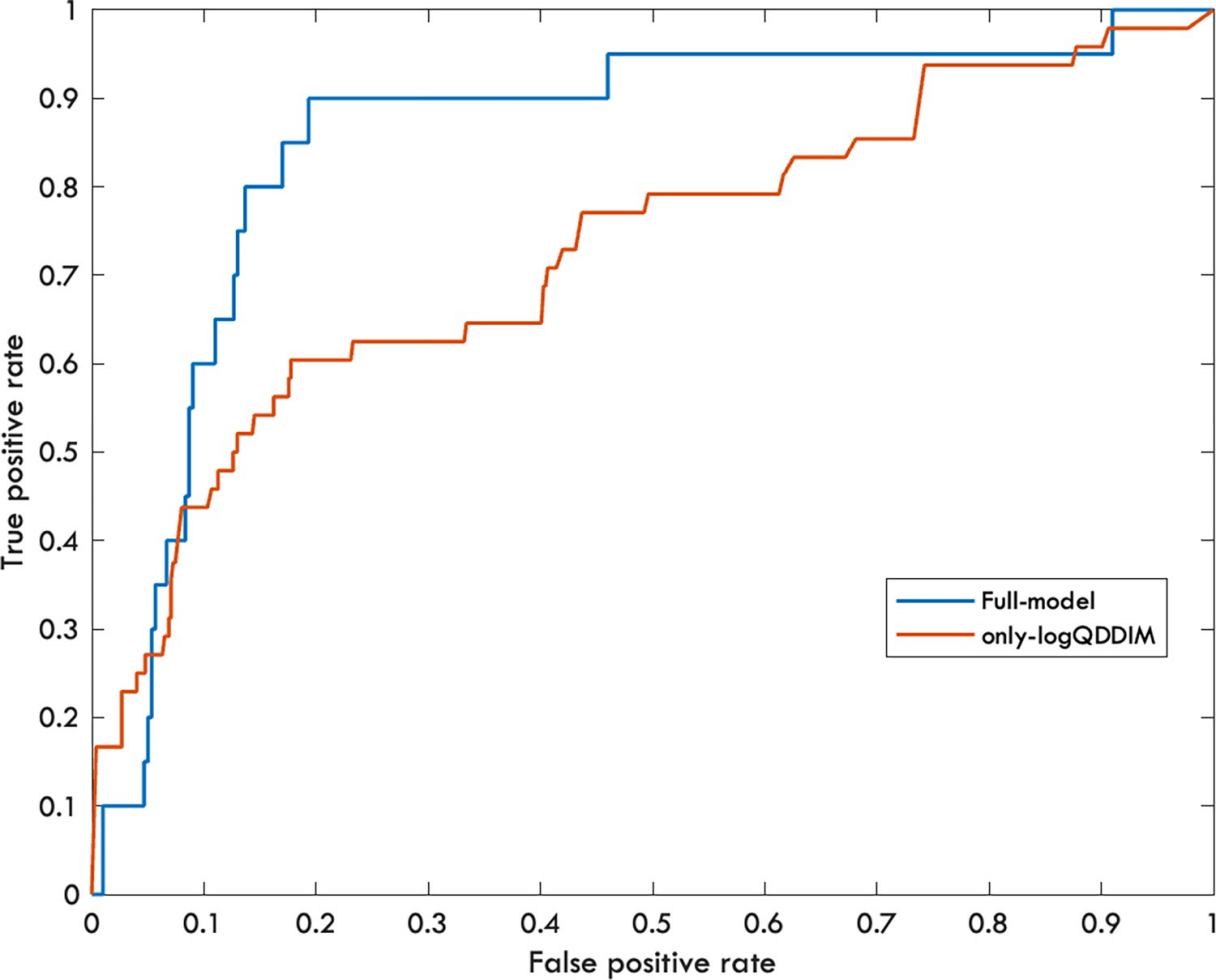
Receiver operating characteristic (ROC) curve for PE diagnosis. Blue curve represents the full model including predictors such as age, gender, disease duration, logQDDIM and CTSI (AUC = 0.85). The red curve stands for the model in which only the logQDDIM was used (AUC = 0.72).

The multiple logistic regression model indicated that the only significant predictor of PE was the logQDDIM (p<0.016, degree-of-freedom: 314). Other predictors were not significant (p>0.05). The area under the curve for the ROC analysis was 0.85 (Fig 2).

Modelling a real-life situation, when the complex model cannot be estimated for each patient, we only analysed the single, most significant predictor, the logQDDIM. The logQDDIM was able to predict the PE on its own as well (p<0.0001, degree-of-freedom = 570, AUC = 0.72).

**Fig 3:**
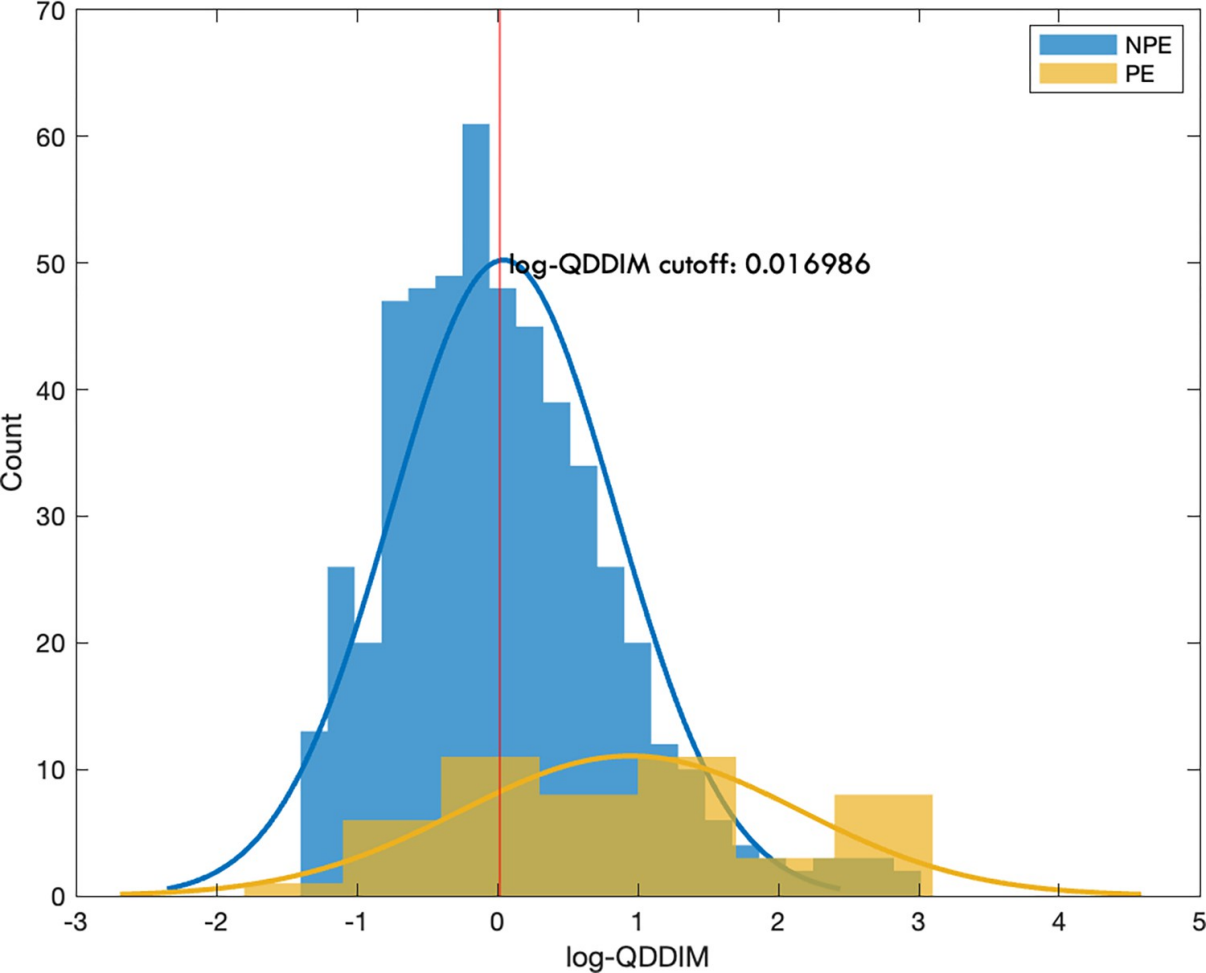
Distribution of the logQDDIM values in patients with (yellow) and without (blue) PE. The vertical red line represents the cut-off value of logQDDIM belonging to 99% sensitivity.

A cut-off value was defined at a high, 99% true positive rate, to decrease the possibility of missing patients with PE (Fig 3). For this the logQDDIM cut-off value was 0.0169, which corresponds to 1.0171 μg/ml serum D-dimer concentration. For 95% true positive rate the cut-off was 0.0273 that corresponds to 1.0277 μg/ml serum D-dimer concentration.

For a 99% sensitivity the cut-off values of logQDDIM was 0.017 that corresponds to 1.0171 μg/ml serum D-dimer concentration. The cut-off value for 95% sensitivity was 0.216 that corresponds to 1.0219 μg/ml serum D-dimer concentration.

**Fig 4:**
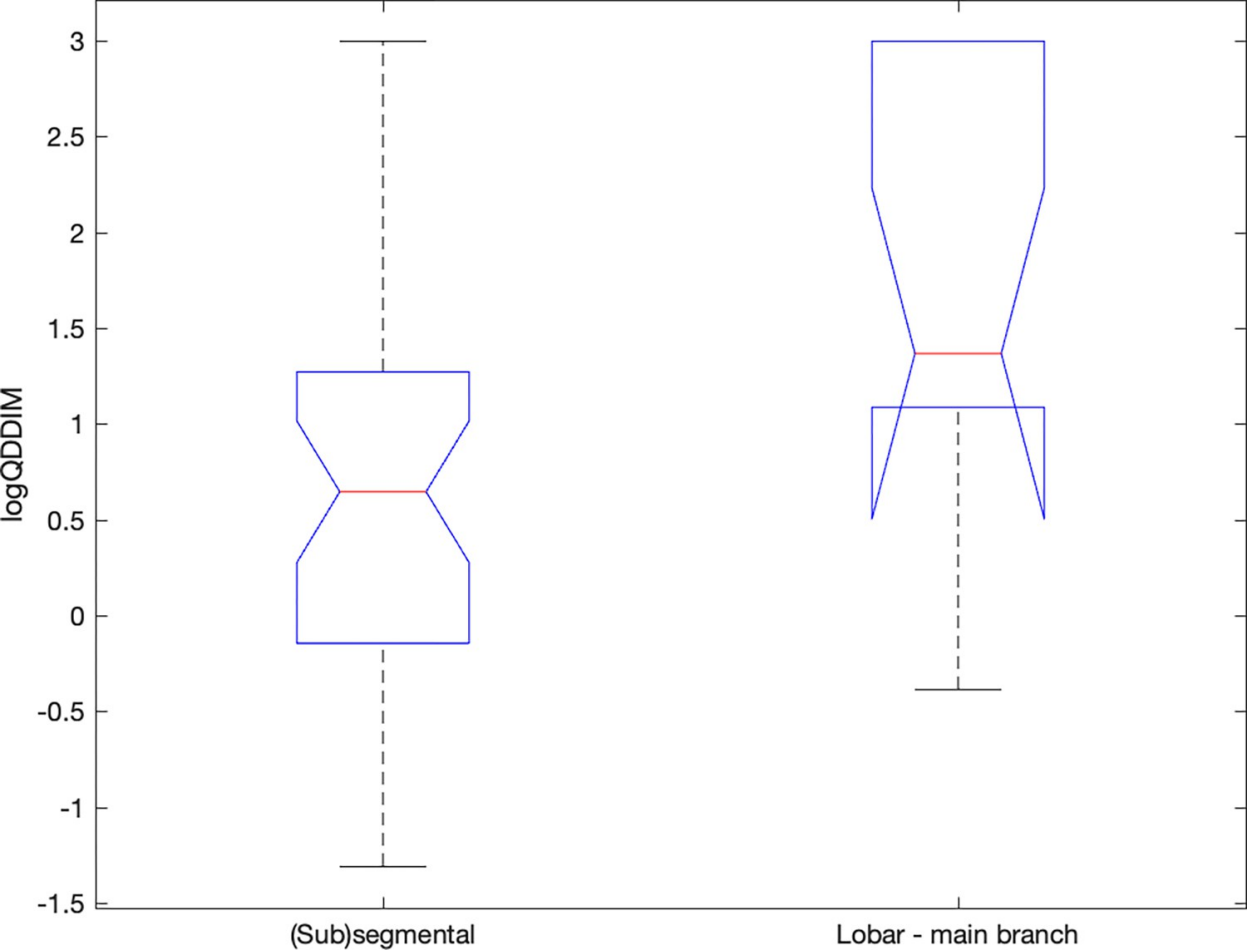
logQDDIM values in the main branch-lobar and in the (sub)segmental PE group. In the (sub)segmental PE group the logQDDIM values were significantly lower. The red lines representing the medians, the box stands for the 25th and 75th percentil and the whiskers extend to the most extreme data points not considered outliers.

Importantly, in patients with lobar or main branch embolus, the logQDDIM was higher when compared to patients with segmental and subsegmental emboli (p<0.05, Fig 4).

**Fig 5:**
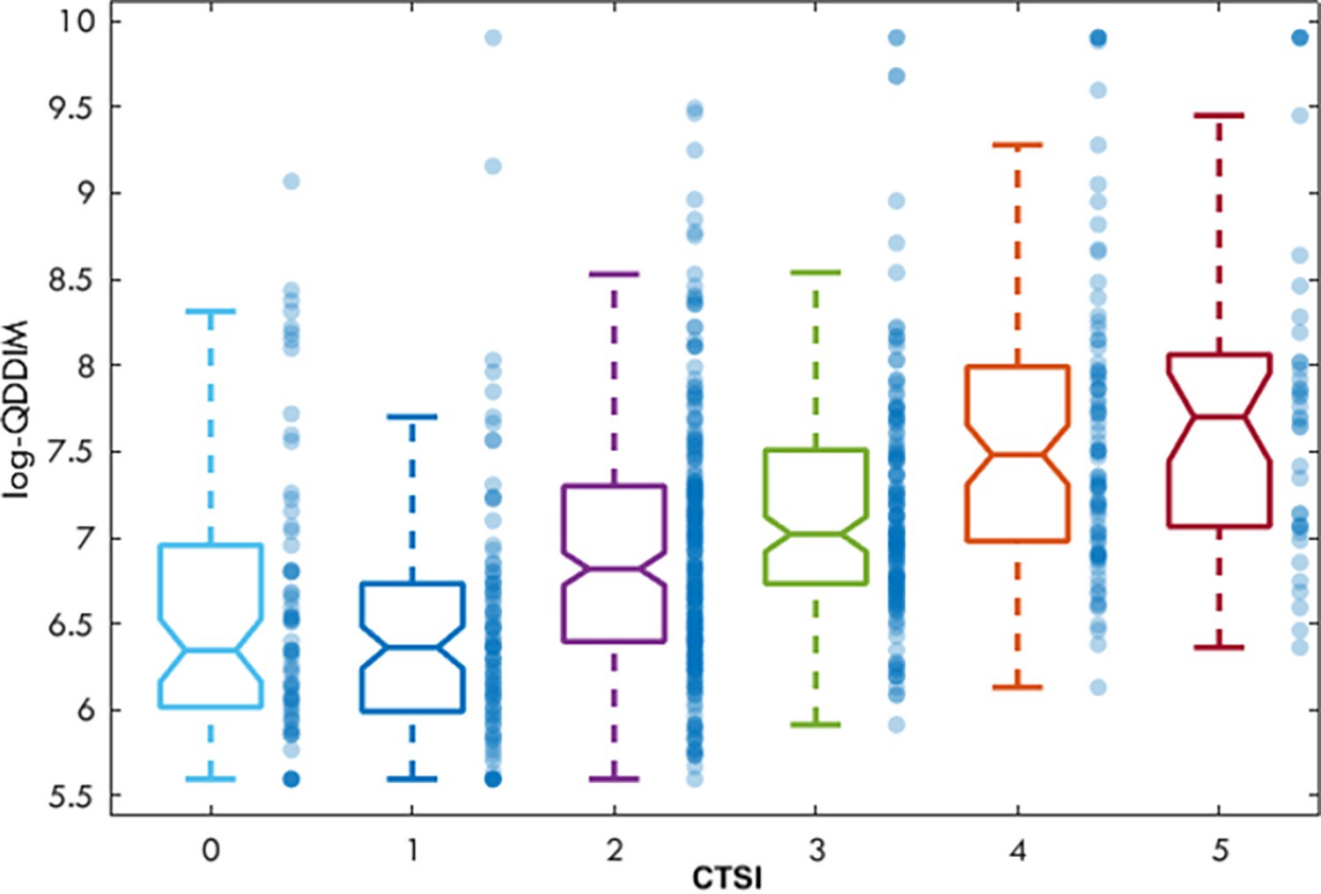
The logQDDIM values in the various CTSI groups. The horizontal lines in the centre of the boxes are representing the medians, the box stands for the 25th and 75th percentil and the whiskers extend to the most extreme data points not considered outliers.

Notably, while the CT severity index was not a significant contributor to PE directly, it correlated positively with D-dimer values, the most significant predictor of PE (Fig 5).

The correlation between the CTSI and D-dimer levels was not significant (R = 0.43, p>0.05).

## Discussion

Our results showed that the prevalence of PE in a large group of COVID-19 patients was 7% at first clinical presentation. The main strength of our investigation compared to other reports [11,12] is that the investigated population was much larger and not only a selected sub-group of outpatients were involved.

Among the investigated possible predictive factors of PE, only D-dimer values showed a strong association with the occurrence of PE. D-dimer is formed when cross-linked fibrin is broken down. Therefore, this process is accompanied by an exponential increase in D-dimer levels if an embolus is present. Blood D-dimer levels correlate with the probability of PE [13,14]. It is well established that PE can be ruled out in patients with low clinical pre-test probability at D-dimer levels of less than 0.5μg/ml [15–17]. A recent study showed that 1μg/ml can be used as a cut-off in patients with moderate clinical probability [18].

COVID-19 increases the risk of thromboembolic complications. Hence, one should not directly generalise the criteria from non-COVID-19 literature to rule out PE. Less stringent pre-test scores might also be related to PE in COVID-19. Our results showed that a blood D-dimer level around 1μg/ml (1.0171μg/ml to be precise) can rule out PE with 99% sensitivity. Furthermore, using this threshold we would miss only a single patient with PE if the small segmental and subsegmental embolization/thrombosis is not considered.

The D-dimer cut-off value in our COVID-19 cohort is very similar to what was reported in the literature earlier [18]. In 100 consecutive CTA examinations of non-COVID out-patients between June and September 2019 we found 19% prevalence of PE, out of which 7% were main branch or lobar. The same analysis of the 85 patients who had D-dimer values available indicated logQDDIM = 0.0947 to have a 99% sensitivity (D-dimer level = 1.1023ug/l). This cut-off would only miss a single patient with subsegmental embolus. While a direct comparison would not be valid, the two D-dimer values are in the same range and similar to those found in the literature.

The high sensitivity of D-dimer to identify COVID-19 patients with large vessel PE is especially important if we consider the clinical relevance of isolated subsegmental PE, since the therapeutic significance of small peripheral lesions is debated [22,23]. Removing small thrombi is considered a normal function of the lung [24]. Moreover, a recent Cochrane review did not find well-designed, randomised, prospective studies on the effectiveness and safety of anticoagulation therapy in isolated subsegmental PE [25].

There is a discrepancy between the occurrence of deep vein thrombosis and PE in COVID-19 that is larger than in the non-COVID-19 population [26]. Based on this observation the peripheral filling defect, which is usually non-obstructive, is more likely to be locally formed thrombi rather than emboli [19]. This was supported by the postmortem pathological findings of thrombotic or thrombo-hemorrhagic microangiopathy in COVID-19 patients [20].

The prevalence of PE is lower in an outpatient setting than in ICU-treated COVID-19 patients. ICU patients are expected to have a longer disease duration. However, disease duration was not a good predictor of PE in this out-patient population. While the pulmonary involvement (CT severity score) was not a significant predictor of PE, logQDDIM values correlated with the severity score (Fig 4).

Medical imaging is the largest artificial source of radiation exposure that adds up to about 0.6mSv/year [27]. With the COVID-19 pandemic and the abundant use of CT it is conceivable that the population radiation dose will increase. Furthermore, it is expected that the mean age of the scanned COVID-19 population is lower than the population scanned before the pandemic. Hence it is very important to perform CTA examinations in patients only if PE cannot be excluded by any other means. By measuring D-dimer levels, we could have reduced the number of CTA examinations in our population by 48.6%.

Another issue to consider about CTA in COVID-19 patients is the contrast material used for imaging. CT contrast agents are known to be associated with acute kidney injury [28]. The strongest risk factor for contrast associated acute kidney injury is pre-existing renal disease [29]. Acute kidney injury is frequently reported among COVID-19 patients [30]. Currently there is only one report on the contrast-associated-kidney-injury in COVID-19 patients needed coronarography [31]. The authors reported that 33% of their patients (14/42) developed contrast associated acute kidney injury. Based on the above mentioned information it is critically important to use the contrast material in COVID-19 patients only when it is unavoidable.

The major weakness of our study is that it was retrospective. However, since more than 85% of the COVID-19 patients went through CTA during the observation period, the selection bias must be rather low. Furthermore, having the clinical pre-test probability between the predictors would have an added value. The virus is evolving with time and vaccination was limited at the time of the study, hence the applicability of the results to the current situation needs further investigations.

## Conclusion

SARS-CoV-2 infection and the developing COVID-19 disease increase the risk of thromboembolic events. While CTA is a sensitive approach to detect PE, in order to reduce the radiation dose, avoid the contrast related complication and the risk of procedure related infection of health-care personnel, a pre-screening method based on plasma D-dimer levels could be very useful. We suggest that plasma serum D-dimer levels of less than 1μg/ml safely exclude PE in COVID-19 outpatients. In this case the CTA examination is not warranted.
